# The role of early diagnosis of emphysematous cystitis: A case report and literature review

**DOI:** 10.1016/j.eucr.2021.101581

**Published:** 2021-01-26

**Authors:** F. Kowalski, J. Adamowicz, J. Jozwicki, D. Grzanka, T. Drewa

**Affiliations:** a Department of Urology, Antoni Jurasz Memorial University Hospital, Bydgoszcz, 85094, Kujavian-Pomeranian Voivodship, Poland; b Department of Pathomorphology, Antoni Jurasz Memorial University Hospital, Bydgoszcz, 85094, Kujavian-Pomeranian Voivodship, Poland; c Collegium Medicum, Nicolaus Copernicus University, Bydgoszcz, 85037, Kujavian-Pomeranian Voivodship, Poland

**Keywords:** Emphysematous cystitis (EC), Salvage cystectomy, Computer tomography (CT), Urinary tract infection (UTI), Inflammation, Infection, Bladder, CT, Computer tomography, ICD, Intensive Care Unit, EC, emphysematous cystitis, UTI, urinary tract infection, VSD, Vascular Surgery Department

## Abstract

Emphysematous cystitis (EC) is a rare entity caused by bacteria, which produce gas filled cysts in the bladder wall. We present a case of EC in a 72-year-old woman admitted to Vascular Surgery Department because of diabetic foot syndrome. During the hospital stay, the patient's general condition deteriorated. CT established EC diagnosis. Surgical treatment was inevitable. Salvage cystectomy was performed. Despite macroscopic removal of necrotic tissues, the condition of the patient didn't improve, 75 days past diagnosis of EC she died due to the multi-organ failure. Prompt diagnosis provided by imaging plays a key role in the treatment of EC.

## Introduction

Emphysematous cystitis (EC) is a rare type of infection characterized by the presence of gas in the bladder wall and lumen. There are a few confirmed risk factors of EC like recurrent urinary tract infections (UTI), neurogenic bladder disorder, atherosclerosis, female gender, but diabetes seems to be the most important, due to its pathogenesis. An accurate diagnosis of the disease gives good results. Our case study presents a patient with EC revealed by computer tomography (CT) scan during hospitalization in the Vascular Surgery Department (VSD) due to diabetic foot syndrome.

## Case presentation

**Fig. 1:**
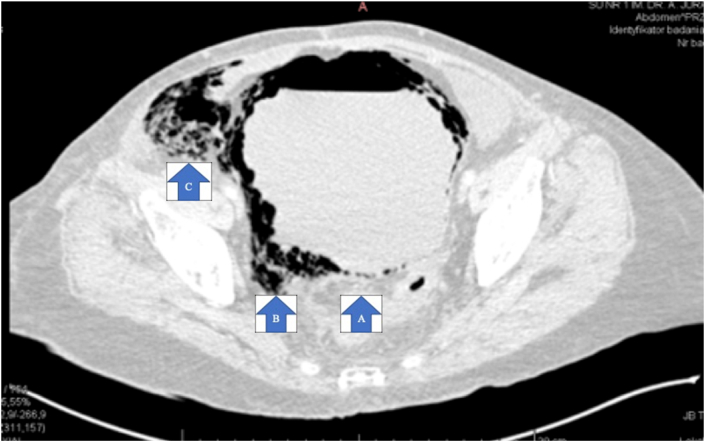
CT scan (transverse plane) showing emphysematous cystitis (A) Bladder lumen with urine and gas (B) Gas bubbles in the bladder wall (C) The inflammatory process passes into right groin (gas bubbles in the right groin).

**Fig. 2:**
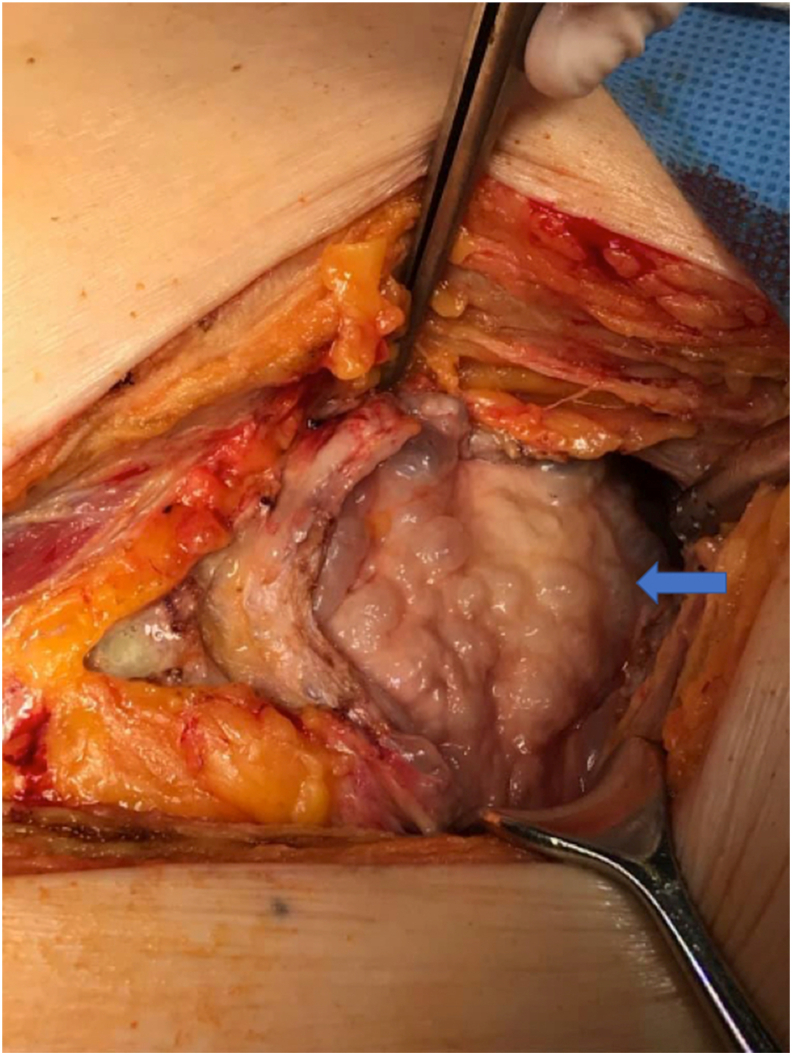
Intraoperative photograph of emphysematous cystitis. Bladder walls are inflated by gas leading to formation of bubbles (arrow) [photo F.Kowalski].

**Fig. 3:**
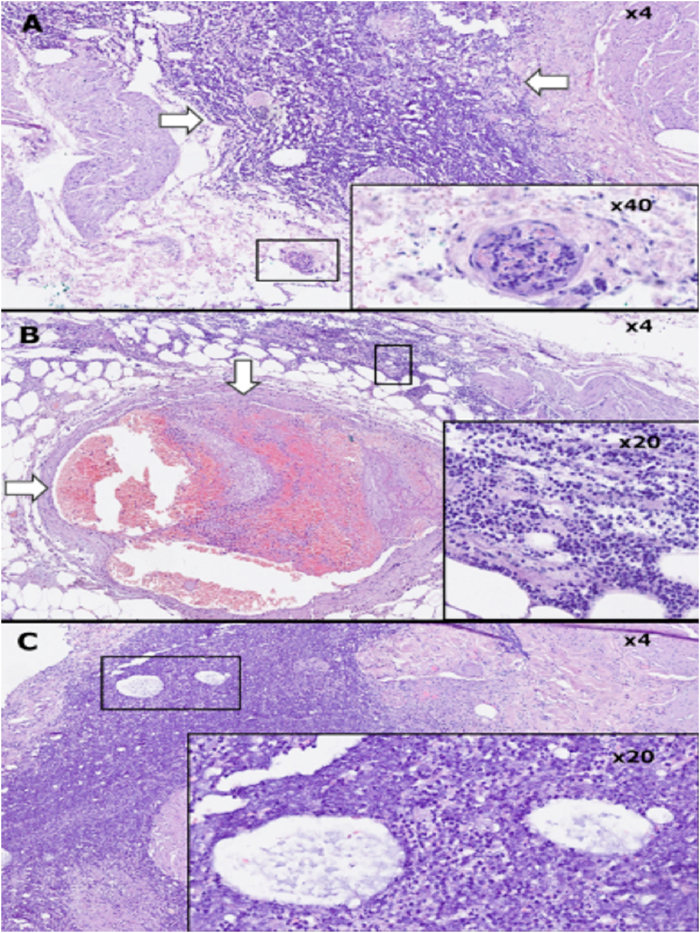
Histopathologic examination revealed features of pyogenic cystitis A. The bladder wall with vast band of acute inflammatory infiltrate near the surface (arrows); frame: thrombus in small superficial blood vessel. B. Thrombus in large blood vessel within perivesical fat (arrows); frame: phlegmonous infiltrate of perivesical fat. C. Frame: dilated lymphatic vessels in superficial layers of bladder wall mimicking pathognomonic cysts [photo J.Jozwicki].

A 72-year-old woman with diabetes, hypertension, atherosclerosis, grade 3 renal failure, treated in the past for ovarian cancer and stricture of the upper, left ureter secondary to the retroperitoneal removal of benign tumor was admitted to the VSD because of diabetic foot syndrome. On the second day of hospitalization the patient underwent arteriography where obstruction of superficial femoral artery was diagnosed. The day after arteriography the patient had fever. Broad-spectrum antibiotics (Vancomycin) were administrated. Laboratory tests showed persistent high levels of glucose (>200 mg/dl); inflammatory parameters were increasing (CRP from 294 to 453 mg/l, WBC from 25000 to 27000/μL, procalcitonin from 0,8–12 ng/ml); anemia was present (Hgb 7,8–9,5 g/dl); worsening of renal failure was observed (creatinine from 2,14 to 3,14 mg/dl). On the 7th day of hospitalization in the VSD, due to the ileus and septic shock syndrome, a CT scan was performed. CT demonstrated massive emphysema of the bladder with expansion to the right groin and colon, causing obstruction of the lower half of colon (Fig. 1) and 700ml of urine and gas in the bladder have been reported. After urological consultation, the patient was catheterized and admitted to the Department of Urology, immediately qualified for urgent laparotomy. During operation we found that most of the bladder walls were destroyed (Fig. 2) and the patient was qualified for salvage cystectomy. Due to the respiratory instability, ureters were looped and nephrostomies were implemented to shorten the time of surgery procedure, the paralytic obstruction was discharged. *Escherichia coli* infection was confirmed in intraoperative bacterial cultures and in urine culture. Pathological report revealed pyogenic cystitis and peritonitis. Histopathologic findings were severe, but not specific for EC, lack of multinucleated giant cells did not meet the criteria for diagnosis of EC (Fig. 3). After the surgery, the patient was admitted to the Intensive Care Department (ICD) due to the acute respiratory failure. During hospitalization in ICD, the patient underwent two laparotomies due to ileus. The first laparotomy revealed peritoneal abscess, which was evacuated and adhesions were released. The second laparotomy revealed ileum necrosis, 30 cm of ileum was resected and a double-barreled stoma was created. In further hospitalization in ICD, amputation of the right lower limb at thigh level was performed due to progressive ischemia. After 46 days in ICD, the patient was dismissed to Urological Department where after 2 days she developed acute respiratory failure secondary to pneumonia and was readmitted to ICD. After 27 days in ICD the patient died because of multi-organ failure.

## Discussion and conclusions

In the first stage of the disease, patients with EC present mild symptoms like in uncomplicated cystitis. This phase of the disease is connected with bacterial colonization. In the second stage of the disease, pneumaturia can occur. There is no clear explanation of occurrence of gas in the EC. Since most of the cases concerned women with poorly controlled diabetes, authors postulated a theory of high glucose concentration in bladder tissues which leads to acid fermentation by *Enterobacteriacae* causing production of hydrogen and carbon dioxide.^1^ However, the occurrence of EC among patients with no glucose metabolism disorders suggests that in exceptional circumstances the bacteria can cause fermentation of proteins as well.^2^ If the second phase of the disease is not given proper treatment, which consists of decompression of the bladder and broad-spectrum antibiotics, EC can spread locally causing dysfunction of nearby organs, and in effect can lead to the collapse of homeostasis and cause sepsis.^3^ Imaging is the most important tool in diagnostic process. Nowadays, CT seems to be the most adequate diagnostic tool allowing to differentiate EC from the bladder fistula and to access local advancement of the disease.^4^

The described case of the female patient was characterized by many, high risk factors of EC. The patient had decompensated diabetes, advanced atherosclerosis, underwent radiotherapy and instrumentation of urinary tract causing recurrent UTI. At the time of the diagnosis, significant local advancement of the infection was disclosed. Such advancement combined with sepsis and ileus significantly worsened the prognosis – the patient needed emergent, extensive surgery procedure which additionally weakened the patient's immune system. In the opinion of most authors of the publications concerning EC, the need for surgery worsens the prognosis of EC and should be kept to a minimum such as decompression of the bladder and removal of necrotic tissues.^5^ In the case under discussion, CT scan was performed in last phase of the disease which delayed decompression of the bladder and proper treatment however the symptoms are nonspecific which make a diagnosis difficult, even pathological report was ambiguous. At the time of the diagnosis, extensive, urgent surgical treatment was imperative which resulted in worse prognosis.

Availability of fast and wide imaging facilitates proper diagnosis of the disease and implementation of treatment including decompression of bladder, targeted antibiotics and diabetes compensation. Imaging plays a key role in diagnosis of EC due to the nonspecific symptoms and pathological results. Patients with UTI symptoms and risk factors of EC like blood supply disorders, anatomical and neurogenic disorders, systemic disorders, should have a basic, immediate imaging (ultrasound, plain film of abdomen) to set a diagnosis in the appropriate moment. Extended imaging should include a CT scan which allows proper identification of local advancement of the disease and recognition of other causes of gas in the bladder. Early diagnosis of EC allows for effective conservative treatment which results in good outcomes.

## Funding sources

This research did not receive any specific grant from funding agencies in the public, commercial, or non-for-profit sectors. The publication was paid for by Nicolaus Copernicus University in Torun.

## Declaration of competing interest

None.
